# Gender and organizational culture in the European Union: situation and prospects

**DOI:** 10.3389/fpsyg.2023.1164516

**Published:** 2023-04-25

**Authors:** Nuria Alonso Gallo, Irene Gutiérrez López

**Affiliations:** ^1^Department of Applied Economics, Faculty of Law and Social Sciences, Rey Juan Carlos University (URJC), Madrid, Spain; ^2^Complutense Institute for International Studies (ICEI) Complutense University of Madrid (UCM), Madrid, Spain; ^3^Department of Business Economics, Rey Juan Carlos University (URJC), Madrid, Spain

**Keywords:** gender equality, organizational culture, glass ceiling, gender parity on boards, European Union law

## Abstract

In recent decades, there has been a massive incorporation of women into the labor market. However, the belief that certain jobs or business functions can be performed better by people of one gender than the other has not allowed for widespread changes in the business culture to achieve effective equality between women and men in companies. Examples of this are unequal access to employment, vertical and horizontal segregation in occupations, wage discrimination, problems in reconciling personal and professional life, or difficulties in accessing management positions in companies (glass ceiling). Other determinants of gender inequalities have been long working hours, as well as the presence of employees, characteristic of European business culture. The progress achieved to date began with the incorporation of women into the labor market under unequal conditions that soon called for the need to establish a regulatory framework to try to eradicate them. The legal status of women in Europe has undoubtedly improved as a result of the development of European regulations, which have been binding in the development of business policies in the Member States and have succeeded in modifying the organizational climate through proposals such as the development of Equality Plans or salary audits. Examples of the most recent legislative initiatives of the European Union on equality that affect business practices are Directive 2022/2041/EC on adequate minimum wages in the European Union or Directive 2022/2381/EC on a better gender balance among directors of listed companies. This study attempts to systematize the changes in the legislation on effective equality between men and women in business and to analyze its effect on organizational culture through the information available in the statistics on gender equality—mainly from the European Union—which gather quantitative and qualitative information on the adaptation of business culture to the new legal framework and the overcoming of gender stereotypes that have been guiding business management in the last decade.

## 1. Introduction

Generally, studies that attempt to analyze the degree of effective equality between men and women in the economy focus on macroeconomic aspects such as the multiple gender gaps in the labor markets or the public policies and legal measures that would be necessary to reduce these differences between men and women. By contrast, in many cases, these inquiries fail to consider the psychological and social conditioning factors that trigger those conducts within companies which can only be overcome with a firm commitment to change in the corporate culture.

It is interesting to note how within each company, there are replicas of complex, dynamic societies, composed of diverse subjects that follow several ethical principles, marking the role of each member that is part of them. Thus, how personal and interpersonal relationships are developed will affect their business success. Organizational culture is responsible for analyzing what are the opinions, norms, and values that are spread within companies, and how they condition the behavior of the staff (Bayón Pérez, 2019). Remarkably, however, in business terms, climate refers to the perception that a company's employees have of its corporate culture. Following (María Jesús, 2016), this notion is defined as “the perception of people, regarding the work context in which they carry out their work, at a given moment in time[...], something that can be managed over time by knowing and activating the levers of improvement of those aspects of working life that most influence attitudes of satisfaction and commitment at work.” By contrast, (Cisneros and La Torre, 2004) consider that “organizational culture should comply with, among other functions, making organizations different, generating a sense of identity, orienting behaviors toward institutional goals, facilitating the company's adaptation to the environment, to learning, to change, and maintaining the internal social system stable.”

Based on a specific culture, the concept of business climate arises from the need to connect the part of purely corporate values and social relations within companies. Therefore, public policies must encourage the highest levels of the business organization to establish those ethical values and equity essential to achieve equality between women and men in terms of employment conditions, salaries, and professional careers. As part of organizations, people's careers are as important as their personal development. In this sense, workers should know in advance what values underpin the companies where they will spend their time and knowledge over a long period of their lives. Chiavenato (2011) highlights that “to know an organization, the first step is to know its culture since being part of it means assimilating it. To live in an organization, to work in it, to take part in its activities and to make a career is to participate intimately in its culture.”

Considering all this, the aim of this article is 2-fold: first, to analyze the different regulatory measures implemented by the European Union (hereinafter EU) and national legislations in the last decades, to promote the incorporation of women into the business world, and to reduce the gaps in employment, salaries, and professional careers. Within this first objective, this article will also try to evaluate the measures' effectiveness based on the following hypothesis: The legal measures established to reduce male hegemony at the highest levels of business management have been effective and may be a driving force for both changing the business culture in terms of gender equality and removing stereotypes about the managerial capacity of women and men. Second, this study will also examine the progress made in the business environment by establishing the current situation in terms of equality between men and women in business in EU countries. It is evident that in recent decades, there has been a massive incorporation of women into the labor market, thus becoming affected stakeholders by everything that has happened in companies. Thus, it is necessary to know what role women play in labor relations, whether they are in an equal position to men, or whether, on the contrary, women find a climate of discrimination in their jobs, which translates into negative results for the whole set of corporate policies. In this article, therefore, it will be shown that by analyzing the position of women in the organizational culture, the dominant culture in companies has been male. Hence, individuals occupy a system that organizes power relations through a relationship of inequality between men and women (Reverter Bañón, 2008), and the business world is no exception to this reality.

## 2. Corporate culture and gender equality

After making an exhaustive selection of the existing academic literature on corporate culture and gender equality, the notion of Social Gender Responsibility (hereinafter SRG) begins to gain a place as it should be deployed to address all the problems that women face in the labor market. Following the state of the earlier question, it is widely known that there is ample theorization of the importance of business culture and ethics in the fight against gender inequalities. Nonetheless, this study will show that the databases and statistics that can support the models proposed and suggest new future practices are practically scarce.

The establishment of gender equality in organizations has long been demanded to be a principal objective of social responsibility actions. Corporate Social Responsibility (hereinafter CSR) can be defined as a form of business management that focuses on considering the impact of its activity on both society and the environment. Yet, some scholars (see Larrieta et al., 2014) claim the necessity of building an SRG system within companies where activities should include a gender perspective, either if these are internal to the company, such as human resource management, occupational health and safety, adaptation to change, and management of environmental impact and natural resources, or external, such as local communities, business partners, suppliers and consumers, human rights, and global ecological issues (Larrieta et al., 2014).

However, women's insertion in new public spaces, such as the workplace, has not exempted them from reproducing the gender patterns imposed (Martínez Méndez, 2018). That is why gender equality should form an essential part of corporate culture even if evidence has shown that in most cases, legislative tools are needed to force companies to adapt their corporate values and intensify their strategies against gender discrimination. Many authors agree on signaling a direct relationship between CSR and the notion of gender mainstreaming. Particularly, they emphasize that if CSR deals with analyzing the links with interest groups such as stakeholders, it consistently needs to be concerned with gender impact. Historically, women have been overlooked in the process of considering these stakeholders (Pearson and Seyfang, 2002). Nonetheless, given the integral part played by workers within these agents, the establishment of systems that respect them is widely needed. Cogently, this can only be executed by eradicating the gender discrimination that still prevails in companies' important issues such as remuneration, family life reconciliation, and women's access possibilities to management positions. As González González (2021) signals, while the culture of a company cannot be legally regulated, social responsibility, ethics, and the requirements of stakeholders may influence the company to comply with certain objectives beyond legal obligations. Thus, it is necessary to identify how companies accept gender equality regulations in the achievement of their business objectives, specifically in soft laws. In this respect, gender-inclusive leadership may provide different perspectives on equity, generate a higher level of philanthropic activities, and affect the quality of CSR initiatives to ultimately allow companies to achieve a great degree of success (Soares et al., 2011).

Historically, the current proposals for equality within CSR may have been aimed at complying exclusively with the “minimum share.” That is, they promote the balanced presence of men and women on the boards of directors of large organizations. By contrast, although this is relevant, it is not unique since they need to consider the rest of the perspectives that range from internal dimensions, where the aspects directly related to business management are situated, to external ones, which directly relate to other aspects such as sustainable development and human rights (Boldó Roda, 2015).

Many authors argue that the strategies of organizations should consider gender equality and take concrete actions in this area. In this sense, promoting equal opportunities in the organization needs to be a leading strategic objective (Bastida, 2009). One of the main hypotheses is to question the current economic model as its goal is still to maximize the wealth of corporate stakeholders while increasing economic and financial probability. Nonetheless, organizations also need to be a driving force for the economic and personal development of the individuals that integrate them (Rumí et al., 2016). Interestingly, different European countries began to foreground the importance of CSR in terms of gender equality and equal opportunities after the Lisbon Treaty (European Economic Community 2007). This resulted in the development of the Green Paper, also known as “Promoting a European Framework for Corporate Social Responsibility” (Commission of the European Communities, 2011). In this publication, the notion of Corporate Social Responsibility and some of its main characteristics are included:

“CSR is a voluntary integration, from the part of the companies, of social and environmental concerns into their business operations and their relationship with stakeholders—Being socially responsible does not only involve fully complying with legal obligations, but also going beyond by investing ‘more' in human capital, the environment, and stakeholder relations.” Furthermore, it also highlights how those companies that pay attention to social aspects, such as gender equality, can improve their results and generate greater economic growth. CSR, therefore, “is not simply an optional aspect that should be added to the company's main activities but rather an element that may affect its own management.” Vacca et al. (2020) indicate that participation in CSR practices proves to be a fundamental factor in a company's survival and success. CSR represents a company's commitment to contribute to economic development and improve social and environmental standards within the European Union and internationally.

Returning to the model based on the maximization of corporate profit, many studies have tried to modify the widely acknowledged business ethics. The business world has long been masculinized, so it is necessary to reflect on how business ethics can help to achieve gender equality in organizations. As the branch of ethics has been established as a discipline that conglomerates the “should-be” dimension, the demands on gender equality should transcend the conditionality to become a formal reality within a business. Cortina Orts (2003) argues that “ethics is broader in scope than law. Law is about avoiding deviant behavior [...] but ethics is about ethos and incorporating into the character of individuals and organizations those habits that can lead to fair decisions.” Considering back the notion of GSR (Kahale Carrillo, 2013), it entails the incorporation of the issue of gender into CSR management to reinterpret the existent relationship between companies and stakeholders through a gender lens. Therefore, GSR is based on a business ethic that is sensitive to the problem of gender discrimination which attempts to address current issues faced by women in the labor market ranging from the sexual division of labor and gender wage gaps to sexual harassment in the workplace. In this way, gender equality becomes one more value of the corporate culture, for it turns into a central part of business management and any work dynamics adopted.

For over three decades now, corporate managers are constantly looking for ways to balance their commitments to the owners of the company and their obligations with the growing group of stakeholders who are constantly claiming both legal and ethical rights (Carroll, 1991). The founder of the pyramid theory, who set out four types of companies' social responsibilities with a pyramid structure, indicated that “social responsibility would only become a reality if a growing number of adults become moral rather than amoral or immoral.” Grosser and Moon (2005) holds that the scope and subsequent application of a correct CSR need to combine the technical processes for measuring and reporting social results with political processes to redefine corporate rights and responsibilities, always considering the different forms of stakeholders' participation.

Business ethics would thus become the perfect tool to provide a universal framework for reflection, regardless of territory and different legal frameworks, which works to solve the conflicts that may arise and suggest good actions beyond legal schemes (Medina-Vicent, 2016). Similarly, the scholar also advocates the need of complementing law with ethics to move toward truly egalitarian societies (Medina-Vicent, 2015).

Although company owners attract a wider interest since they are responsible for taking decisions, research also focuses on identifying what groups of stakeholders can demand gender equality and the consequences of a balanced number of men and women in a company's economic power. As cited by Solimene et al. (2017), diversity on the shareholder board produces positive effects due to different knowledge, skills, experiences, ideas, and behaviors; thus, a heterogeneous board can better meet the requirements of the company's stakeholders. While some instances may not reflect the positive effects that gender equality could have on business results and the regulations do not sanction companies, one compelling case would be to receive pressure from the part of stakeholders to make organizations include concrete actions within their strategic objectives. de Luis Carnicer et al. (2011) allude to several scholars that advocate a current trend in business management that centers on increasing the benefit for the whole society. In this way, this new management considers an equal distribution of benefits and opportunities for all the groups involved in the activities of a company. Furthermore, they point out that even though women should not be considered as stakeholders since they make up 50% of the population, they will form part of all possible agents affected by companies, such as workers, suppliers, customers, managers, and owners, and it is necessary to acknowledge that their inferiority within stakeholders is highly visible. If companies wish to maintain their position in society, then, it will be necessary to move from strategic business management to one based on active listening to their environment, while maintaining reciprocal relationships with their stakeholders. The theory of stakeholders (Freeman, 1984) highlights that ethics and morality should play an active role in the business world and, particularly, in the achievement of gender equality. Freeman claims that managers should make corporate decisions by respecting the welfare of stakeholders rather than using them to achieve a corporate end. In this respect, the scholar claims that companies should advocate for equal opportunities that allow female employees to have the same conditions as their male counterparts. Thus, any action that promotes inequality between women and men within the corporate structure would not be legitimate. Departing from this ethical model which promotes a reciprocal relationship between business organization and stakeholders, the foundational basis for the integration of a gender perspective within a company is established.

In conclusion, it is necessary to consider Amelia Valcárcel's observation that behind the individuals who will be part of a board of directors or business management, there is an underlying ideology that overlooks the candidate's experience, training, and credentials. Cogently, this perpetuates gender stereotypes to place the capabilities of men in leadership positions (Valcárcel, 2007). Taking this into account, the necessity of implementing legal measures that force organizations to balance the power rather than locate it in the hands of those who, despite not having the necessary skills, comply with the biological identity held as superior.

## 3. Methodology

This article uses a mixed-method approach since it examines quantitative and qualitative information regarding women's situation in companies and is different from the 27 EU Member States according to their current configuration. It has been emphasized that one of this study's objectives is to explore the relationship between existing regulations on gender equality in the workplace and the development of corporate culture. This would help to evaluate whether situations of discrimination against women take place in the climate in which people work. Furthermore, the target is also to verify whether legislative advances have improved the situation in recent decades by urging companies to develop new organizational culture practices that promote equality between women and men at all workplace levels. In this way, the analysis developed in this article has a temporal dimension which started in the last 30 years with the IV Conference on Women (United Nations, 1995) and is considered an important turning point in this area.

First, a legal analysis will be made by reviewing the progress made in recent years in terms of gender equality and the labor market. This scrutiny would help to both analyze and comprehend the explanatory memorandum of any legislative proposal and to evaluate the repercussions and effectiveness of the law. Specifically, this study will be focused on two main aspects: First, the inspection of the main international and community texts that highlight both the discrimination suffered by women in the labor market and the importance of scheduling gender equality as a main objective for sustainable economic development; second, the enumeration of the different European Directives which has given significant advances to gender equality in the business environment. The primary source has been the EUR-Lex platform (Publications Office of the European Union, 2022), which gathered all EU legislative texts, grouped them, and updated them at different stages of the legislative process. In this section, the regulatory impact of the countries that have implemented laws on gender parity on boards of directors has been considered to determine whether they have encouraged the promotion of women to the highest levels of business and decision-making.

This will be followed by an analysis of the information available from different statistical sources in the EU. Particularly, this examination would be useful to obtain a diagnosis of the current situation, its evolution, and evidence of the effectiveness of the legal measures implemented. It is important to emphasize that these issues have not been the current priority of the legislative and political agendas of the Member States. Consequently, there are no *ad hoc* European statistics on business culture or climate from a gender perspective.^1^ That is why a selection of relevant information from different statistics was developed to help make an approximation in three stages:

The first stage corresponds to the analysis of all the variables that show the changes in the incorporation and quality of women's employment in the national labor markets. Specifically, this scrutiny has been carried out through data collected in the Labor Force Survey (hereafter LFS) (European Statistical Office, Eurostat 2022), a sample survey that collects quarterly and annual data over a wide period with the participation of people over the age of 15 and people outside the labor force.

The second stage will investigate the variables related to the professional development of women, especially in their access to management positions, whether middle management or executive. As this information is not available in the LFS, the analysis has been carried out through the European Working Conditions Survey (hereinafter EWCS) and the Gender Equality Index of EIGE.

The final stage will develop an in-depth analysis of qualitative indicators and opinion surveys. This examination would show the maintenance of gender stereotypes as well as other elements of corporate culture through the thoughts of employees in their jobs. In this case, the information has been obtained both from the EWCS and from specific surveys prepared by Eurobarometer.^2^

## 4. Regulatory review

### 4.1. Current EU regulations affecting the labor force

**Figure 1:**
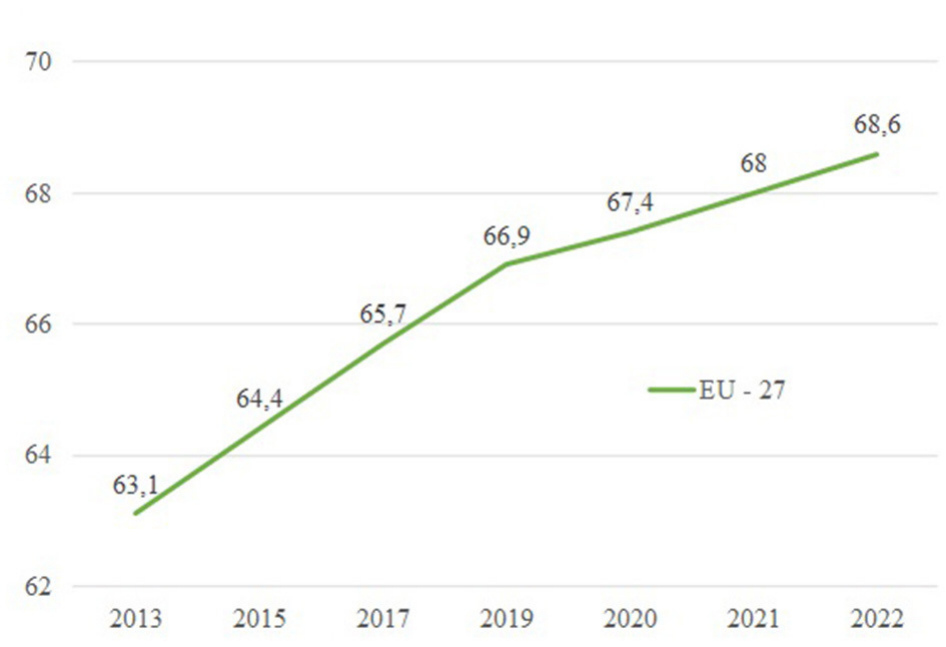
Gender equality index EU-27. Source: Own elaboration based on data from Gender Equality Index, EIGE^3^.

Before offering an overview of the gender equality EU regulations, it is necessary to define its current situation. For this purpose, the Gender Equality Index of the past 10 years developed by the European Institute for Gender Equality (hereafter EIGE) would be used. This index is a measurement tool that synthesizes the complexity of gender equality by looking at its multiple dimensions with six core domains: work, money, knowledge, time, power, and health; particularly, this tool measures the progress of gender equality in the EU, makes visible those areas in need of improvement, and assists policymakers in designing more effective gender equality measures. Figure 1, for instance, depicts how progress in gender equality has been slow due to decision-making improvements as it will be subsequently analyzed in-depth.

In 2013, the index stood at 63.10 points, and as the graph shows, approximately a decade later, it has only increased by less than 5 points. Considering that the index gives a score from 1 to 100 to the Member States, in which 100 would imply the achievement of full equality for men and women, the index backs up the argument that there is still work to do. Some studies confirm that with the current EIGE data, it will take almost three generations to achieve gender equality. Interestingly, if the 68.80 points corresponding to 2022 are itemized, it can be seen how the domain of power is the one with the lowest score, at 57.20 points. This domain directly refers to gender equality both in the political sphere and in companies' decision-making bodies which results from organizational culture.^3^

**Figure 2:**
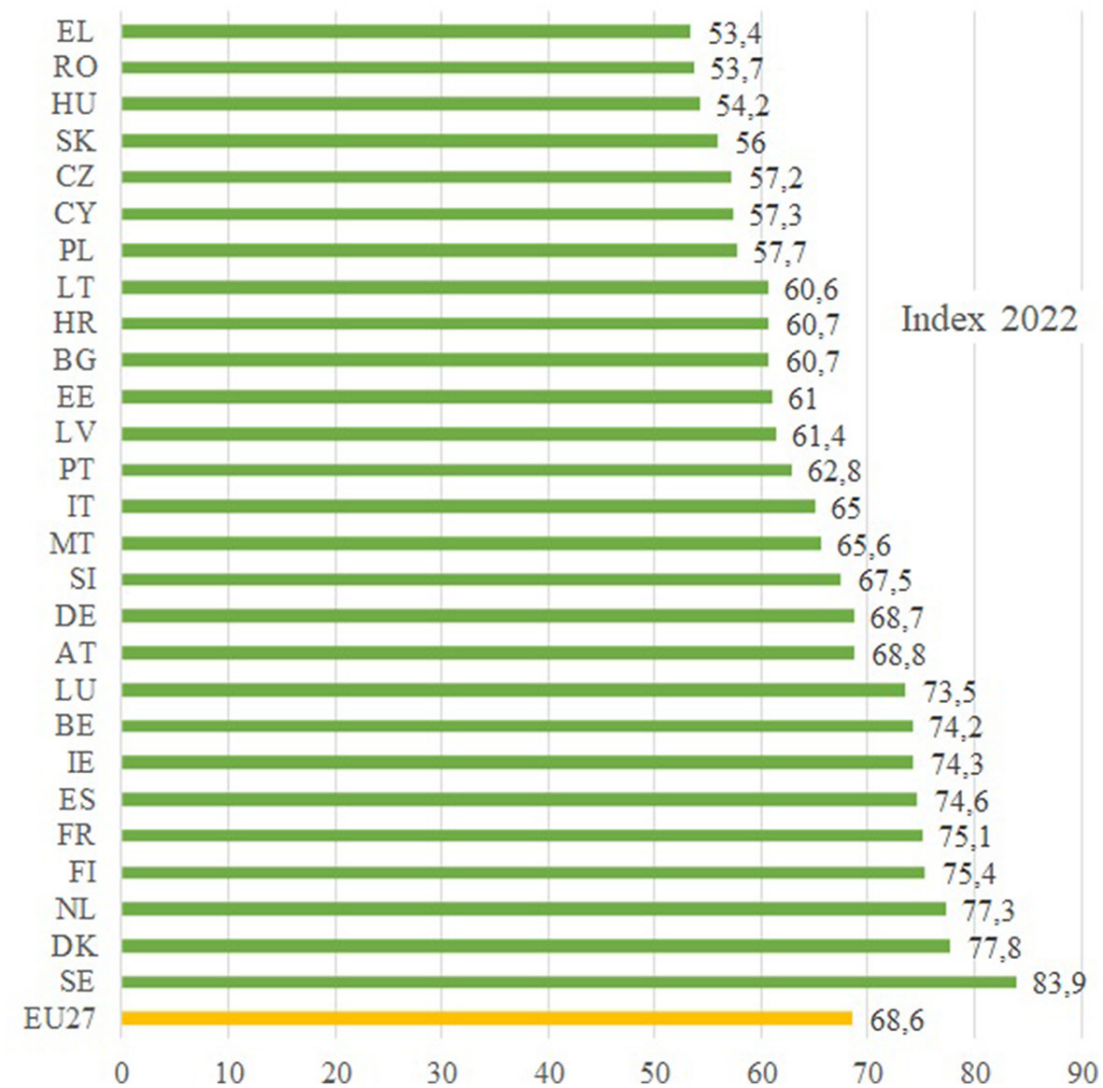
Gender equality index by countries, 2022. Source: Own elaboration based on data from Gender Equality Index, EIGE.

A detailed analysis of this index by country is shown in Figure 2, which reveals that the 16 Member States are below the EU average while the remaining 11 grew faster in gender equality, thus reducing their distance from the EU average. In this respect, the countries' central domain of power suggests that there is still a latent gender imbalance in large companies' decision-making processes. Nonetheless, the proportion of women on board in some of the EU's largest listed companies reached a historic high of 32% in April 2022 due to changes in countries with binding legislation.

Another key indicator of the situation of women in the labor market is the Global Gender Gap Report (World Economic Forum, 2022). This document, respectively, compares the status and the evolution of gender parity in four key categories: economic participation and opportunity, educational attainment, health and survival, and political empowerment. According to the 2022 latest report, we will need around 132 years to reach complete gender parity. Of the four sub-indexes that compose the report, we developed an in-depth exploration of the gender gap in leadership by industry section. Interestingly, the analysis shows that the percentage of women hired in leadership roles has steadily increased from 33.3% in 2016 to 36.9% in 2022. Nonetheless, while the rate of women in leadership has escalated over time, women have not been recruited equally in all industries. On average, more women have been hired for leadership positions in industries where they already have a high representation. Remarkably, however, only specific industries, such as non-governmental organizations (47%), education (46%), and personal services and welfare (45%), have levels close to gender parity in leadership. Directly opposing these rates are industries such as energy (20%), manufacturing (19%), and infrastructure (16%).

Taking all these into consideration, it could be argued that the data presented are not coincidental since they illustrate that women still suffer inequality in many areas, in particular, the data reflect that the largely proclaimed equality between men and women is still an unattained goal, which, as we will show, is more prevalent in legal texts than in the wider society. This reflection in legal and gender terms illuminates that, despite the social acceptance of the rules and the involvement of public authorities in achieving equality, some important aspects that regulate these laws are not fully effective. This is because its breach only generates a series of social or academic criticisms, but not a real sanction.

Over the past three decades, the European Commission (hereinafter EC) and the Council (from now on EUCO) have generated some plans, measures, and directives to favor equality between men and women in business for all Member States. Nonetheless, one of the weaknesses that have been found in this regulatory framework is that the laws fail to explain how they can be correctly implemented and binding for all Member States. As it will be shown, the EU, the United Nations, organizations, and political groups have implemented certain actions to increase the participation of women in business decision-making. While this has produced certain improvements in some countries, particularly those with privileged economic and educational development, they are not enough.

Still, the measures proposed at the legal level are dependent on the willingness and ethics of those who are in charge of putting them into action. Medina-Vicent (2016) highlights that one of the problems in the effective implementation of gender equality in companies may lie in the fact that the gender issue has been incorporated into European regulations not from the search for equality as a basic human value but from a purely economic necessity. In this way, the commission may have been requiring companies to exploit the expertise of women not as a way of promoting social justice but rather as an objective to attain European economic efficiency.

An important turning point in the achievement of gender equality was the IV Conference on Women (United Nations, 1995). However, before delving into the main advances of this declaration, which is an international precedent since it included the concept of gender discrimination for the first time, some important elements need to be foregrounded. For instance, the ILO convention no. 11 (International Labour Organization, 1958) concerning Discrimination in Respect of Employment and Occupation, and the Convention on the Elimination of All Forms of Discrimination against Women (United Nations General Assembly, 1981), where the importance of the Charter of the United Nations (United, 1945) is exposed. From the former, it is interesting to consider article 11.1: “States Parties shall take all appropriate measures to eliminate discrimination against women in the field of employment in order to ensure, on a basis of equality of men and women, the same rights, in particular […] (b) The right to the same employment opportunities, including the application of the same criteria for selection in matters of employment; (c) The right to free choice of profession and employment, the right to promotion, job security and all benefits and conditions of service and the right to receive vocational training and retraining, including apprenticeships, advanced vocational training and recurrent training; (d) The right to equal remuneration, including benefits, and to equal treatment in respect of work of equal value, as well as equality of treatment in the evaluation of the quality of work; (e) The right to social security, particularly in cases of retirement, unemployment, sickness, invalidity and old age and other incapacity to work, as well as the right to paid leave; […]” Following the chronological order of this text, it is also necessary to mention the proposal of the United Nations Commission on the Status of Women. This was the main international body devoted to fostering gender equality and women's empowerment. In 1987, this regulatory body developed a work program following the guidelines held by the III Conference on Women (United Nations, 1985). These codes established several priority areas, which include the promotion of equality in both economic and social participation and decision-making.

A conference that stands out from previous World Conferences is the Beijing Declaration (United Nations, 1995), particularly it was important since this conference introduced the novel concept of gender mainstreaming. This notion serves to indicate the need to incorporate a gender perspective in all policies and programs so that before a decision is taken, the effects on women and men are, respectively, analyzed. Most important, perhaps, is that the report of this conference was unanimously adopted by 189 countries, and thus established certain strategic objectives in favor of women's empowerment.

For this article, chapter F: Women and the Economy has been explored in-depth. This chapter reflects how the absent presence of women in economic decisions affects not only their public realm but also their private one. In this way, the entire society is completely disturbed by the unequal economic structure between men and women. As such, it formulates certain objectives which should be attained by national governments which would be responsible for: establishing actions such as guaranteeing women's equal pay rights with men (equal pay for equal work or work of equal value), effectively enforcing laws against sex discrimination in the labor market, adopting measures that protect women's biological status in terms of reproductive function, such as not hiring or firing women because of pregnancy or breastfeeding, developing positive measures to enable women to participate on equal terms, reviewing national tax systems to eliminate any possible discrimination against women, continuing to work on measuring unpaid work, and modifying employment policies to make it possible to share family responsibilities, among many others.

The year 1999 saw the coming into force of the Treaty of Amsterdam (European Economic Community, 1997) which is relevant for the establishment of equality between men and women in the EU, particularly in the labor market. One of the most important articles in this respect is Article 141 which includes the establishment of work for equal value and the possibility of adopting positive discrimination measures. Article 141.1: “Each Member State shall ensure that the principle of equal pay for male and female workers for equal work or work of equal value is applied.” 2- “For the purpose of this Article, ‘pay' means the ordinary basic or minimum wage or salary and any other consideration, whether in cash or in kind, which the worker receives directly or indirectly, in respect of his employment, from his employer. Equal pay without discrimination based on sex means: (a) that pay for the same work at piece rates shall be calculated based on the same unit of measurement; (b) that pay for work at time rates shall be the same for the same job. 3- The Council, acting in accordance with the procedure referred to in Article 251, and after consulting the Economic and Social Committee, shall adopt measures to ensure the application of the principle of equal opportunities and equal treatment of men and women in matters of employment and occupation, including the principle of equal pay for equal work or work of equal value. 4- With a view to ensuring full equality in practice between men and women in working life, the principle of equal treatment shall not prevent any Member State from maintaining or adopting measures providing for specific advantages in order to make it easier for the underrepresented sex to pursue a vocational activity or to prevent or compensate for disadvantages in professional careers.” The interest in this article derives from the fact that it heavily influenced the subsequent development of Community Directives (hereinafter Dir.). That is, the Treaty of Amsterdam laid the foundations so that the EU could develop several measures to strengthen the binding and enforceability of the Member States' national law; particularly, our analysis centers around those affecting the labor market, and, for this purpose, we established five categories indicated in Table 1 that were subsequently compared with the statistics of the counties in which these measures have been materialized to effectively promote equality:

**Table 1:** European legislation on equality.

**Category**	**Legislation**
Incorporation of women into the labor market.	→ Dir.2000/78/EC, on the establishment of a general framework for equal treatment in employment and occupation. → Dir.2006/54/EC, on the implementation of the principle of equal opportunities and equal treatment of men and women in matters of employment and occupation. → Regulation. (EC) no. 1922/2006 on establishing a European Institute for Gender Equality.
Type of workday.	→ Dir.97/81/EC, on the Framework Agreement on part-time work. → Dir.2008/104/EC, on temporary agency work.
Equal pay.	→ Dir.2019/1152/EU, on transparent and predictable working conditions in the European Union. → Dir. 2022/2041/EU, on adequate minimum wages in the European Union. → Action Plan EU 2017–2019 tackling the gender pay gap between men and women.
Work-life balance/parental leave.	→ Dir.92/85/EEC on the introduction of measures to encourage improvements in the safety and health at work of pregnant workers and workers who have recently given birth or are breastfeeding. → Dir.2010/41/EU on the application of the principle of equal treatment between men and women engaged in an activity in a self-employed capacity. → Dir.2019/1158 EU on work-life balance for parents and carers.
Professional development/economic power.	→ Council Recommendation 96/694 on the balanced participation of women and men in the decision-making process. → Dir.2022/2381/EU, on improving the gender balance among directors of listed companies and related measures.

Source: Own elaboration based on legal texts included in EUR-Lex.

Interestingly, the elaboration of these legal texts has evolved. For instance, the Dir.2000/78/EC on the establishment of a general framework for equal treatment in employment and occupation failed to mention the category of sex as discriminatory. Even so, it laid a foundation that could be applied equally to the unequal treatment of women in the workplace. Similarly, the text also includes a definition of the concepts of direct and indirect discrimination, which are key in the fight against gender inequality among women workers.

Regarding Dir. 2006/54/EC on the implementation of the principle of equal opportunities and equal treatment of men and women in matters of employment and occupation, it should be noted that it established major amendments to other texts that were previously approved (Council Dir. 76/207/EEC of 9 February 1976 and Council Dir. 86/378/EEC of 24 July 1986). The reason behind the amendments was 2-fold: (1) to conflate in a single text the main aspects and provisions relating to equal treatment between men and women in the labor market; and (2) to include new developments arising from the case law of the European Court of Justice. In this text, the Council states that it is important to thoroughly specify what society understands by “work for equal value,” a crucial element for the study of the gender pay gap. However, when the directive is analyzed in-depth, it is not farfetched to suggest that the discrimination suffered by women in terms of pay and working conditions largely comes from the lack of work–life balance. That is why the 2006/54/EC is committed to the implementation of positive action measures, and in its last considerations, it states the need to create effective sanctions to comply with equal treatment between men and women: 35) “Member States should provide for effective, proportionate and dissuasive penalties for breaches of the obligations under this Directive.”

It is also necessary to consider the Directives and Proposed Directives that have come into force in the last 3 years. The Dir.2019/1152/EU on transparent and predictable working conditions in the European Union points out that regardless of the type and duration of the employment relationship, workers are entitled to fair and equitable treatment in terms of working conditions.

The proposal for Dir.2021/0050 (COD), to strengthen the application of the principle of equal pay for equal work or work of equal value between men and women through pay transparency and enforcement mechanisms, is still in progress although the first reading took place in the council. In particular, the impulse behind this proposal lies in demanding that equal pay should be guaranteed in all Member States since the EU gender pay gap is currently approximately 14%, which would lead to a poorer quality of life for women in future. Although this requirement is already included in previous directives, it has not been met. Thus, the EU is in constant need to introduce new legal provisions that promote effective measures with which the States should comply.

The recent Dir.2022/2041/EU, on adequate minimum wages in the European Union, comes from a legislative procedure initiated in 2020. This procedure recognizes that women belong to a group that suffers multiple forms of discrimination, and therefore, they are more likely to be minimum or lower-paid workers than other groups. Given the lack of representation of women in low-paid jobs, the improvement of minimum wages would contribute to gender equality, in particular, reducing the gender and pension pay gap would bring women, and their families, out of poverty while promoting sustainable economic growth in the Union. Furthermore, it also emphasizes that the crisis caused by the COVID-19 pandemic has had a significant impact on the service and small business sector, which has a higher proportion of women as precarious wage earners.

Finally, it is also worthy of consideration that the Dir.2022/2381/EU improved the gender balance among directors of listed companies and related measures. This directive has been vetoed by Germany for 10 years, but still, the EC has been able to set the target that, by 2027, at least 40% of board members should be women. However, the aim of this directive is not only to increase the representation of women on boards of directors but also to help attract female talent to the company and ensure a greater presence of women at all levels of management and in the workforce.

### 4.2. Women on the boards of directors: the case of five member states

Although data suggest that approximately 60% of EU graduates are women, they are underrepresented in decision-making processes in the economic sphere in general and, particularly, in the business area. That is, Corporate Europe is still an area dominated by men since only 13% of senior management positions are held by women. This is because the boards of directors usually assert that this is the place where the most important economic decisions of a company should be made. Remarkably, however, one of the main characteristics of all the boards of directors in the Member States is also the lack of female representation. In this sense, it could be asserted that this prevalent absence of women in companies' management bodies remains one of the main unsolved gender gaps in the EU.

Still, it could not be assumed that these alarming data are something natural and inevitable. Very recently, legislative measures have been established to force companies to change this scenario of inequality. To date, one of the best tools developed has been the colloquially called quota system. This system has been adopted, in different forms, by only five states, but regardless of this fact, it has proven to be effective in terms of balancing the presence of men and women in economic power. Nonetheless, it has also shown that as the decision of its application does not impose a sanction in some countries such as Spain and the Netherlands, it is less effective in achieving quota targets in comparison to those states that apply sanctions for not complying with this balanced representation. Thus, it can be argued that the gender quota system is an effective measure to increase the presence of women on the boards of directors (Kirsch, 2021).

For this article, we have conducted an analysis of the characteristics and implementation of gender parity laws on boards of directors in five of the pioneering countries, namely Belgium, France, Spain, Italy, and the Netherlands. Thus, Table 2 shows the following data: the current situation of women on national boards of directors, the minimums established in the standard, whether in addition to these targets for the representation of each sex, there are other practices, such as recommendations in the Corporate Governance Codes (hereinafter CGC), and ultimately, the characteristics of these measures, specifically their duration, and whether they have penalized companies that have not complied with the target set.

**Table 2:** Board parity legislation.

**Country**	**% Women's participation in boards 2022-B1**	**Minimum % of women**	**CGC Recommendations**	**Duration**	**Sanction**
Belgium	37.1%	33% applicable to executive and non-executive directors in state-owned and listed companies.	The 2009 CGC recommends that the composition of a board should be determined based on gender diversity.	Permanent	Open seat
France	46.3%	40% applicable to non-executive directors of large listed and unlisted companies.	Recommendation containing the same fees as in the 2011 Act, applicable to all board members (Amended in the 2020 revision by the requirements of the NFRD).	Permanent	Open seat
Spain	34.7%	40% applicable to both executive and non-executive directors in state-owned companies with 250 or more employees.	CGC Recommendation 15 on gender diversity. In the 2020 revision, a 40% quota is established.	Permanent	No incentives for companies that comply with it.
Italy	39.6%	40% for listed companies and state-owned enterprises. Applicable to management boards and supervisory boards.	The 2011 CGC includes reference to the gender composition of the board. In 2020, it is amended to specify what composition the board should have.	Temporary. In 2020 it was extended for six more offices.	Fines for companies that do not comply with the quota (from €100,000 to €1,000,000).
Netherland	39.5%	30% on the boards of directors and supervisory boards of large companies.	Diversity clauses in the 2009 Dutch CGC, applicable to both executives and non-executives. Voluntary charter with targets for more women in management.	Temporary. A new law was approved in 2021 (not yet in force)	Open seat

Source: Own elaboration based on EIGE data and national legal texts.

Interestingly, the countries that initially established temporary measures have been extending the deadline as the expiration date was coming, even raising the minimum percentages to achieve. Regarding the applicable sanctions, the concept of the open seat is worthy of consideration. This notion refers to the fact that vacant positions on the board can only be filled by a person of the underrepresented sex, otherwise, the appointment will be null and void.

Gender balance in economic power is part of the Sustainable Development Goals of the 2030 Agenda (United Nations 2015) adopted by the United Nations, which aims to improve the quality of life of all people. Goal 5 called “Achieve gender equality and empower all women and girls” is the consequence of the fact that even though women have made significant progress in decision-making positions, their representation is still far from parity. Within the goals of this objective, we find the following in reference to our study: Goal 5.5 “Ensure women's full and effective participation and equal opportunities for leadership at all decision-making levels in political, economic, and public life,” Goal 5.c “Adopt and strengthen sound policies and enforceable laws to promote gender equality and the empowerment of all women and girls at all levels.”

Generally, the participation of women in companies has increased and minimum share proponents argue that diversity will be beneficial for improving equity and business efficiency. Yet, the positions of both top management and boards of directors remain almost an entirely male territory. Cultural norms regarding the prevailing gender roles in a society have a significant impact on the degree to which women are represented in a country's highest decision-making bodies (Holst and Kirsch, 2016).(Holst y Kirsch 2016).

## 5. Statistical analysis

The analysis of corporate culture in gender equality from a quantitative perspective is an intricate issue, particularly when it involves the examination of all the foundational values of corporate culture which try to provide a comprehensible path for all the people who compose the company and daily guidelines that maintain gender stereotypes. Thus, it is necessary to question to what extent this institutional framework perpetuates the belief that certain business functions are better performed by people of one gender than the other. Normally, these functions are ascribed to certain stereotypical values commonly attributed to the male gender, such as competitiveness, violence, fighting spirit, and leadership, which are considered the most suitable for the business environment.

The field of business has been an eminently masculine world in which the incorporation of women into the labor market, not only as employees but also as entrepreneurs, has been relatively recent. Consequently, this has produced a conflict of harmony between the traditional values of the business culture and the new reality of the presence of women in this field. This fact has ultimately led to the appearance of real barriers for women in this area such as unequal access to employment, wage discrimination, vertical and horizontal segregation in sectors and occupations, problems in reconciling personal and professional life, and obstacles to professional development.

As it has been said elsewhere, social, and legal advances to achieve effective equality between men and women in the EU countries prompted a significant reduction in the gaps in the labor markets. Nonetheless, to completely overcome this situation, corporate culture needs to be adapted to the new social situation by refuting the values based on gender stereotypes that currently characterize corporate management. Similarly, if organizations want to both increase and improve the employment rates of women in them, gender equity needs to be a crucial aspect of the culture and the identity of the organization (Marulanda et al., 2019).

The quantitative analysis of the business climate and other organizational elements is not simple, for it involves qualitative elements that are difficult to capture in observable variables. By contrast, at the European level, statistics on business culture that allow for analyzing and monitoring changes in organizations^4^ are absent. Thus, examinations of these elements need to be carried out through gender equality labor statistics and opinion surveys.

The analysis of the evolution and current situation of the business climate and business culture in the EU that this article presents has been carried out in three stages which depend on two elements. In the former, they are largely contingent on the availability of qualitative information. In the latter, they are also determined by our analytical criteria on the range of quantitative and qualitative variables, time series, and cross-section, which will help to develop a thoroughly accurate diagnosis of the situation.

### 5.1. Situation of the labor market in the EU

To characterize the culture of EU^5^ companies in terms of gender equality, it is first necessary to offer an overview of the incorporation of women into the labor markets and their socio-labor situation. Thus, Table 3 shows a synthesis of the most relevant variables with the widest period allowed by the time series available from Eurostat's Labour Force Survey (LFS). Although the data available started in 2005 and ended in 2021, initially, we thought that the year 2021 would be affected by the disruption in the series caused by COVID-19, but the analysis of the 2019 and 2020 data shows that the pandemic does not imply a disruption of the series in 2021. Considering this, we have extracted the following conclusions:

**Table 3:** Labor market conditions for women.

	**Employment rate**		**Employment gap**		**Unemployment gap**		**Part-time employment % of the total and gap**				**Gender pay gap**	
	**Women**		**W-M**		**W-M**		**Women**		**W-M**			
	2005	2021	2005	2021	2005	2021	2005	2021	2005	2021	2006	2020
AT	61,1	68,1	12,6	8,6	0,4	−0,1	39,3	49,2	5,7	10,5	25,5	18,9
BE	53,8	61,8	14,5	6,9	1,8	−0,8	40,4	39,5	7,1	10,4	9,5	5,3
BG	51,7	64,2	8,3	7,8	−0,5	−0,5	2,3	1,8	1,5	1,3	12,4	12,7
CY	58,4	65,3	20,8	11,4	2,1	0,6	13,2	12,7	3,2	7,8	21,8	9,0
CZ	56,3	67,1	17,0	14,2	3,4	1,1	8,0	9,6	1,6	2,5	23,4	16,4
DE	59,6	72,2	11,7	7,1	−0,6	−0,8	43,4	47,4	6,9	10,6	22,7	18,3
DK	71,9	72,6	7,9	5,8	0,8	0,1	32,6	33,5	11,7	15,2	17,6	13,9
EE	63,1	72,4	3,6	3,2	−2,3	−1,3	9,1	16,9	4,5	7,6	29,8	21,1
EL	46,0	48,2	27,4	18,2	9,3	7,6	9,1	12,5	2,2	5,0	20,7	
ES	51,8	57,9	23,3	9,6	4,8	3,6	23,4	22,3	4,4	6,3	17,9	9,4
FI	66,5	71,7	3,8	1,9	0,4	−1,2	18,2	23,2	8,6	11,0	21,3	16,7
FR	58,4	64,5	10,9	5,6	1,5	−0,2	30,3	27,4	5,6	7,6	15,4	15,8
HR	48,6	58,6	13,1	9,6	2,4	0,7	10,7	5,8	5,4	3,7		11,2
HU	51,0	68,2	12,1	9,7	0,4	0,4	5,6	6,7	2,4	2,7	14,4	17,2
IE	58,3	65,5	18,6	8,8	−0,7	−0,3		29,6		11,0	17,2	
IT	45,4	49,4	24,5	17,7	3,8	1,9	25,5	31,5	4,3	8,4	4,4	4,2
LT	59,6	71,9	6,8	1,0	0,4	−1,0	8,8	7,6	5,1	4,3	17,1	13,0
LU	53,7	66,0	19,6	6,6	2,3	0,7	38,2	30,9	2,4	7,0	10,7	0,7
LV	58,2	68,0	8,2	3,9	−0,1	−1,9	9,7	10,0	5,6	5,6	15,1	22,3
MT	33,4	67,3	40,1	15,4	2,1	−0,7	20,2	18,5	4,1	5,4	5,2	10,0
NL	63,0	76,6	15,1	7,0	1,8	0,5	74,7	65,0	21,6	22,5	23,6	14,2
PL	46,8	63,8	12,1	13,0	2,6	0,0	13,3	7,6	7,0	3,3	7,5	4,5
PT	61,6	67,7	11,7	5,0	2,0	0,5	13,3	9,1	3,8	4,7	8,4	11,4
RO	51,5	52,5	12,2	18,6	−1,3	−0,9	9,2	3,0	9,1	4,1	7,8	2,4
SE	70,4	73,3	4,0	4,1	−0,2	0,5	39,2	29,7	10,3	12,0	16,5	11,2
SI	61,3	68,1	9,1	6,4	1,0	1,1	9,8	12,8	6,1	6,2	8,0	3,1
SK	50,9	65,6	13,7	7,7	1,7	0,3	3,9	4,6	1,2	1,8	25,8	15,8
**EU27**	**54,7**	**63,4**	**15,0**	**9,9**	**1,8**	**0,6**	**28,3**	**28,8**	**6,3**	**8,1**	**15,5**	**13,0**

Source: Own elaboration from Eurostat, Labour Force Survey.

In terms of women's employment rates, in 2005, there were a significant number of EU countries with female employment rates below 50% or slightly above that figure. The case of Malta is particularly noteworthy, with a female employment rate of 33.4%, the lowest in the EU, and 37 points below the employment rate in Sweden, which was the country with the highest incorporation of women into the labor market. In addition, the average employment rate in the EU was 54.7% and the deviation of the countries from this average figure was very high, which shows the great heterogeneity of the labor markets at that time. Regarding the 2021 data, the number of countries with female employment rates close to or below 50% has fallen dramatically, the EU average has risen by almost 10 points to 63.4% and the dispersion between countries has narrowed, although Greece and Italy are in a worse situation than they were in 2005.

Similarly, while the employment gap between men and women in 2005 was approximately 15 percentage points in nine countries, and in the case of Malta, this figure exceeded 40 points, in 2021, only four countries had an employment gap of more than 15 points, considering that the average gap in the European Union has fallen substantially. Yet, unemployment gaps between men and women are positive. That is, unemployment is higher for women in most countries in both 2005 and 2021, and although a general reduction is observed, the gaps in Greece and Spain are still very high.

Another of the definitive variables for assessing the incorporation of women into the labor market is part-time employment. Traditionally, Northern European countries have had a much higher incorporation of women into the labor market than the rest of the countries. Nonetheless, these high rates are of part-time employment which implies that women did not have economic independence. Thus, they were not fully part of the organization because they were relegated to the background, for they have a reduced working day and no career expectations. That is why the data in Table 3 shows high rates of part-time employment in some countries such as the Netherlands, with 74.7% of women's employment in 2005, or Sweden, Belgium, Germany, and Luxembourg. At the same time, the part-time employment gap was very low, which means that men mostly had full-time jobs and relegated part-time jobs to women. By contrast, in 2021, the proportions of part-time employment of women and the differences between part-time employment of women and men have not been significantly reduced, and by contrast, they have even risen in some countries such as Germany, Denmark, Finland, and Italy.

As far as wage gaps^6^ are concerned, in 2006, they were higher than 15 points in most countries, including countries with low employment gaps such as Sweden, Finland, Denmark, Estonia, Germany, and Austria. Between 2006 and 2020, a reduction in the wage gap was observed in most countries and very significantly in Luxembourg, Cyprus, Spain, and Poland; however, there was an increase in the wage gap in Latvia, Portugal, Malta, and Hungary.

**Figure 3:**
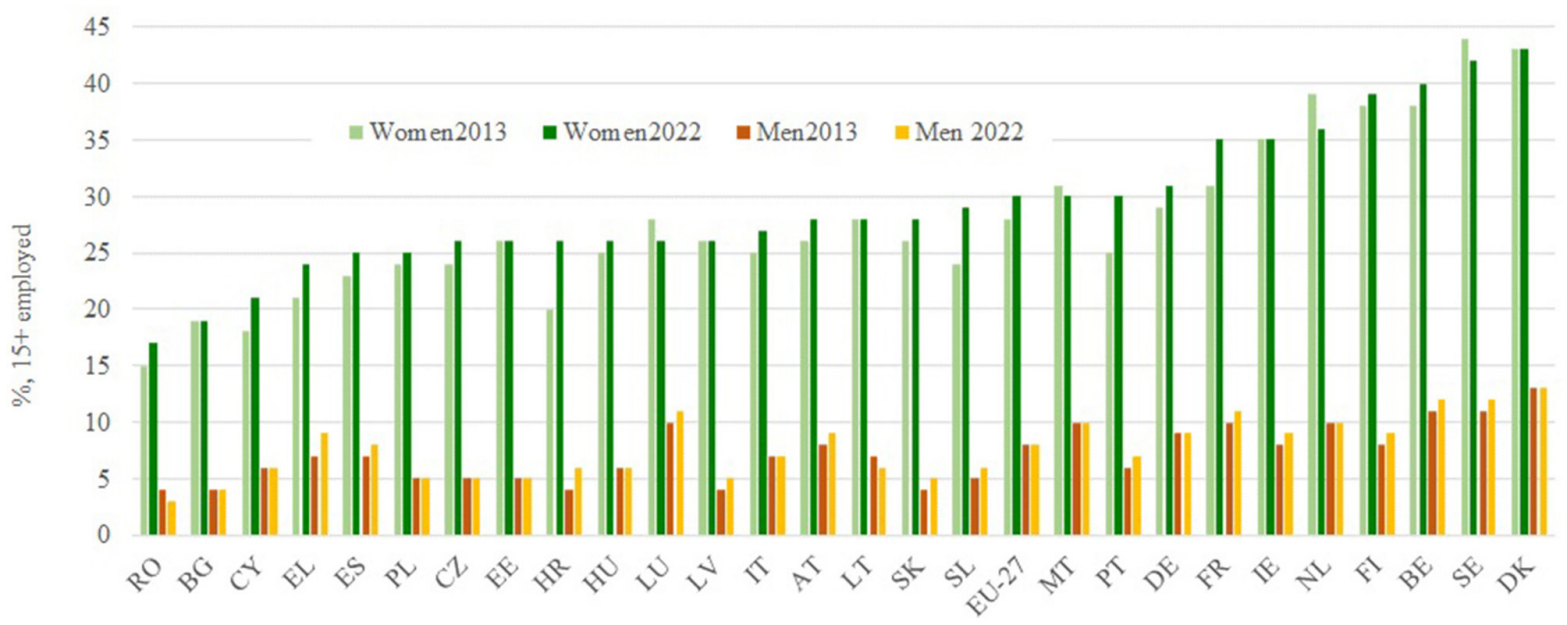
Employed people in Education, Human Health, and Social Work activities (%, 15+ employed). Source: Gender Equality Index, EIGE.

Finally, it is necessary to take into consideration the segregation of employment in specific economic sectors, particularly, those related to education and healthcare. Traditionally, the employment channel for women in all those activities was related to “unpaid reproductive work” performed by them in their homes. This has resulted in the depreciation in labor terms of a good part of the occupations within these productive sectors, and it has manifested in the form of lower salaries. In this way, Figure 3 shows data on the share of employment of women and men over 15 years of age in education, health, and social work activities. In the years 2013 and 2022, respectively, the data are conclusive: except for Romania, Bulgaria, and Cyprus, more than 25% of women in the EU are employed in productive sectors typically occupied mostly by them, and in the case of Denmark, Sweden, Belgium, Finland, the Netherlands, Ireland, and France, this figure exceeds 35%. However, in the case of men, only more than 10% are employed in these sectors in three or four countries. While the data on the evolution between 2013 and 2022 do not show a generalized pattern, both decreases and increases in the employment intensity of women in the sector are discernible.

Therefore, the labor market statistics at the European level reveal that there is greater incorporation of women into the labor market. Nonetheless, although there has been a reduction in the problems of discrimination in terms of working hours and wage gaps, the differences between women and men continue to be generalized, and in many countries, at high levels, especially in terms of the segregation of employment in productive sectors.

### 5.2. Economic power and the glass ceiling

Apart from the situation of women in the EU labor market, it is also interesting to consider the variables strictly related to both the economic power in the firm and the professional development of women. Especially relevant in this respect are the continued segregation of occupations in the workplace that women must face and the issue of the glass ceiling, which have, respectively, been for many years not only on the national but also on the European agenda.

**Figure 4:**
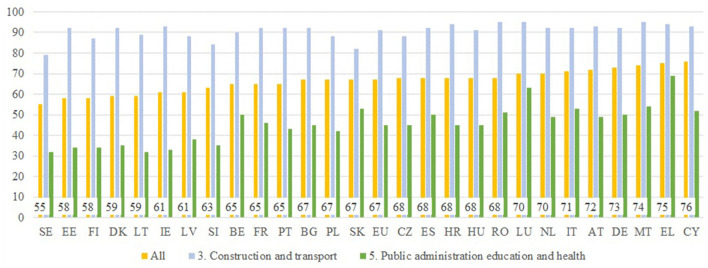
Is your immediate boss a man? Source: Own elaboration from EWCS (2015).

In this regard, the first approximation to the state of the question is found in the results of the 2015 European Working Conditions Survey (EWCS).^7^ Figure 4 summarizes the responses on the gender of the immediate boss in all economic sectors (the figure is shown in the graph) and in clearly masculinized and feminized productive sectors, in particular, it should be noted that in all productive sectors, the proportion of male managers exceeds 50% in all countries. The lowest level is Sweden, followed by Estonia, Finland, Denmark, and Lithuania, as countries with the highest proportion of female middle managers, but still clearly below 50%. In several countries such as Cyprus, Greece, Malta, Germany, Austria, Italy, the Netherlands, and Luxembourg, the proportion of men in middle management or executive positions is 70% or higher. Regarding the distribution of the proportion of male managers in productive sectors in the employment of men and women, data connected to “the construction and transportation” group as a masculinized sector and “the public administration, education, and health” group as a feminized sector. In the case of the “construction and transport” group, the lowest proportion of male managers is in Sweden with 79%, and in a significant number of countries, such as Malta, Romania, Greece, Cyprus, Germany, Austria, Italy, the Netherlands, and Luxembourg, the proportion of male managers exceeds 90%. Respectively, in the “public administration, education, and health” group, the higher proportion of women in employment places the proportion of male managers at around 50%. There are a significant number of countries, including Sweden, Estonia, Finland, Denmark, Lithuania, Ireland, Latvia, and Slovenia, in which the percentage of female managers is higher than that of male managers. As far as the other productive sectors that are not included in the graph are concerned, for instance, the “agriculture and industry” sector has a proportion of male managers similar to that of construction and transportation, and the “commerce and hotel and catering” and “financial and other services” sectors present figures around the average of the “total sectors.”

**Figure 5:**
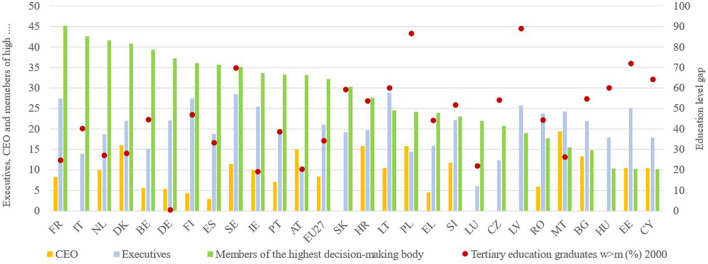
The proportion of women among chief executive officers (CEO), executives, and members of the highest decision-making body of the largest nationally registered companies listed on the national stock exchange 2022. Source: Own elaboration from EIGE.

As shown below, Figure 5 displays the 2022 data for the proportion of women among Chief Executive Officers (CEOs), female executives, and members of the decision-making body of the largest registered companies which are listed on the national stock exchange. Considering the data for CEOs, the proportion of women is nearly zero in most of the countries as opposed to the countries with higher participation of women such as Malta, Poland, Slovakia, Austria, and Denmark with barely 15%. In the case of the team of executives working with the CEO in large, listed companies, the representation of women is higher since it is a lower rank than that of the CEO and equally heterogeneous in the context of EU countries. Yet, Lithuania and Sweden stand out with nearly 29% of female executives and, at the bottom of the distribution, Luxembourg with 6% of women compared to 94% of men.

The third variable included in Figure 5 is the proportion of women in the highest decision-making bodies of large, listed companies at the national level. In general terms, this variable shows the least unbalanced ratio between women and men. In one-third of the countries, the proportion of women exceeds 35%, and in France, Italy, the Netherlands, and Denmark, it surpasses 40%, which, although not equal, would be far from the underrepresentation of the proportion of female CEOs. It is also noteworthy that there is no relationship between female CEOs or executives and those who are part of the highest decision-making body in the different countries. That is, for the time being, no contagion phenomenon favoring the increase of female CEOs or executives has been observed in countries where more than 40% of the members of the highest decision-making body are women and in reverse.

**Figure 6:**
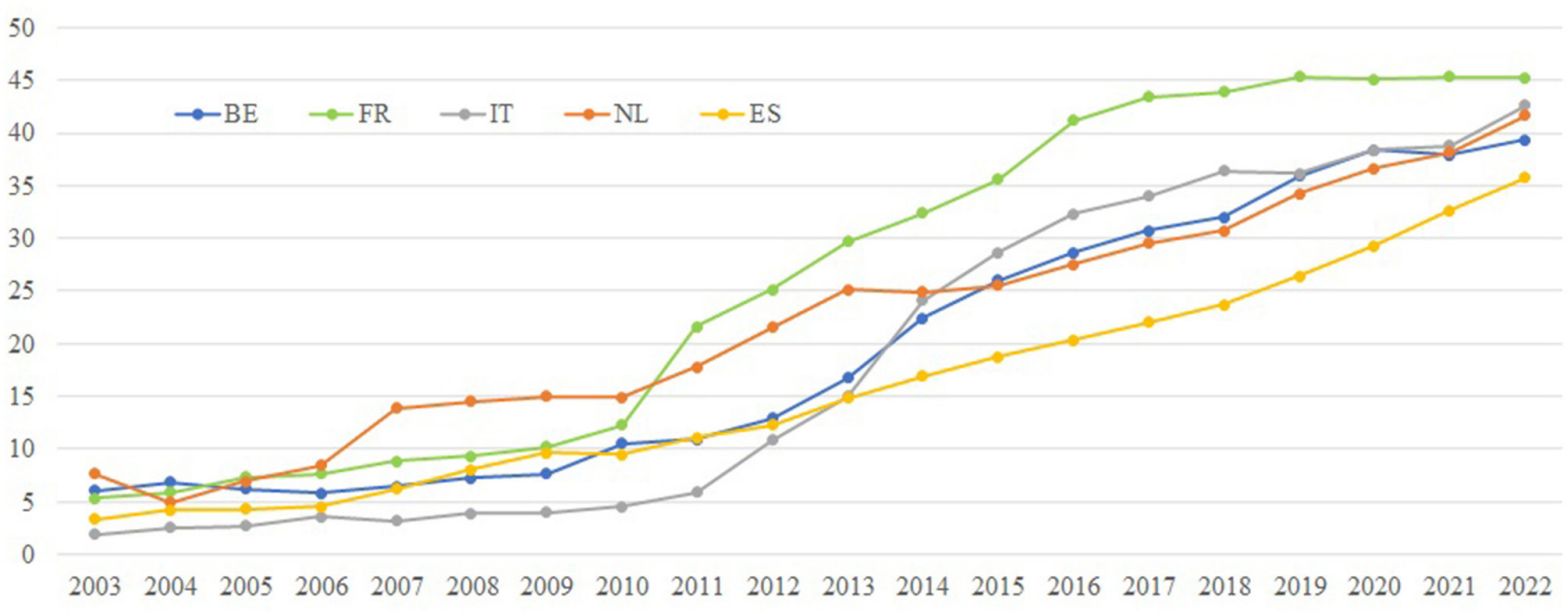
The proportion of women among members of the highest decision-making body of the largest nationally registered companies listed on the national stock exchange and education level gap. Source: Own elaboration from EIGE and Eurostat.

In relation to this increased representation of women in the highest decision-making body, we hypothesize that the breakthrough that has occurred since 2010 may be related to the strong legal and real impetus of the European institutions in achieving the Beijing goals. Precisely, this is the main reason behind the establishment of the European Institute for Gender Equality (EIGE) in 2010 which was aimed to strengthen and promote gender equality throughout the EU. To illustrate this idea, Figure 6 shows the time series of the proportion of women among the members of the highest decision-making bodies of the largest listed companies at a national level, particularly those countries that have adopted legal measures for the equal representation of men and women on boards of directors (the highest decision-making body) between 2009 and 2011, namely Belgium, France, Spain, Italy, and the Netherlands. Even though there are minor differences in terms of the time at which the increase in women's representation begins, the intensity, and the final level reached, as can be seen in the case of Spain (more gradual and less level reached) and Italy (rapid and intense rise from 2011), the pattern of behavior is common to all the countries. That is, the representation of women is almost zero in the early 2000s, then, there is a change in trend around 2010 and a final level of more than 35%. The tertiary education gap has been included in the Figure 5, making evident the higher percentage of women with tertiary education and especially the notable absolute separation between training and executive positions.

**Figure 7:**
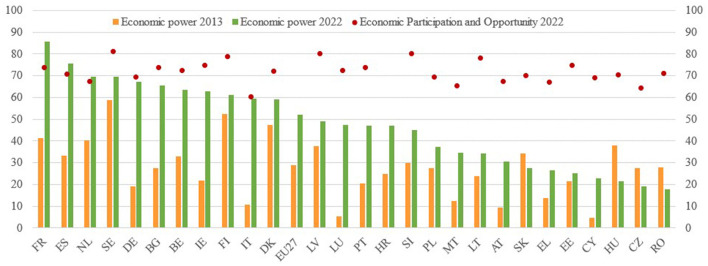
Gender equality index 2013–2022 subdomain economic power. Source: EIGE and WEF.

The gender equality indexes, both the Gender Equality Index (GEI) of EIGE and the Global Gender Gap (GGG) of the World Economic Forum (WEF), are composed of several sub-indexes that study specific areas of gender equality, particularly interesting are the subdomains on Economic Power in GEI and Economic Participation in GGG. That is why Figure 7 shows the sub-index on the Economic Power of the GEI between the years 2013 and 2022. Interestingly, this graph reveals how the countries that have made the most progress in achieving equality of economic power are not those that started from a better situation at the beginning of the series, but those that have established legal mechanisms to advance parity in the Boards of Directors.

Alternatively, in the GGG, within the four sub-indices that comprise the report, the section on gender gaps in leadership by industry has been thoroughly analyzed. More specifically, the examination has focused on the section on gender gaps in leadership by industry, where we see that the proportion of women hired in leadership roles has experienced a steady increase, from 33.3% in 2016 to 36.9% in 2022. However, while the proportion of women in leadership has been increasing over time, women have not been recruited equally in all industries. On average, more women have been hired for leadership positions in industries where they were already highly represented. The report ranks it as the second biggest challenge to equality. Based on the 102 countries analyzed, the remaining gap to be closed in this sub-index is 40%. At the current rate of progress, this gap will close in 151 years, representing several generations to parity.

In sum, the data reveal that progress is slow and inequality levels remain very high. However, it is necessary to highlight the evident but significant progress in the presence of women on the boards of directors of companies that have established affirmative action measures.

### 5.3. Qualitative indicators and opinion surveys

To complement the previous analyses, it is also necessary to develop an examination of the results obtained in surveys on gender discrimination and equality carried out by different EU institutions. Although the surveys do not directly ask about elements of corporate culture, they reveal how different stereotypes and discriminatory elements remain. Interestingly, these results can be deployed as an illustration of the actions that may be carried out in future to improve the corporate culture on gender equality.

Stereotypes that respond to women's specialization in caregiving tasks and their lack of professional ambition and men's position as providers of the household have been considered. Table 4 collects the percentage of affirmative responses to the questions posed in the 2015 Eurobarometer about Gender Equality, with highlighted cells corresponding to those with a high percentage of responses. Interestingly, in all three questions, the percentage of affirmative responses is very heterogeneous between countries while the stereotype is more widespread among women than among men. The first question is “All in all family life suffers when the mother has a full-time job,” and it is the one with the highest percentage of affirmative answers, especially among women and men in Hungary, Bulgaria, and Cyprus. In the case of the second question which is “A father must put his career ahead of looking after his young child,” Hungary is the country with the strongest stereotype between men and women. Ultimately, the answer to the question “Women are less willing than men to make a career for themselves,” has the lowest affirmative answers in most countries with the highest affirmative values given by Hungarian, Bulgarian, and Romanian women.

**Table 4:** Stereotypes about women at work.

	**All in all family life suffers when the mother has a full-time job**		**A father must put his career ahead of looking after his young child**		**Women are less willing than men to make a career for themselves**	
	**Women**	**Men**	**Women**	**Men**	**Women**	**Men**
AT	38,7	31,3	7,2	9,9	10,7	12,3
BE	18,8	11,9	3,6	2,9	5,1	3,1
BG	46,4	43,1	14,3	20,9	16,1	13,8
CY	43,8	43,8	3,6	2,8	4,6	5,9
CZ	27,8	18,5	7,2	8,8	9,8	6,0
DE	31,2	26,1	5,6	5,5	6,3	4,7
DK	13,7	9,2	4,6	3,6	8,3	4,7
EE	27,1	23,4	5,3	4,5	5,8	3,7
EL	40,0	39,0	7,8	8,1	4,2	9,4
ES	39,8	27,7	9,4	9,3	4,6	4,8
FI	6,6	5,1	5,6	5,7	3,6	1,8
FR	24,9	16,8	4,3	3,5	2,6	3,3
HR	27,5	26,7	4,3	5,5	5,9	8,5
HU	47,5	41,4	20,3	20,4	17,2	14,3
IE	24,0	19,1	8,4	9,7	9,1	6,0
IT	26,4	23,2	9,0	8,3	10,6	8,6
LT	37,3	27,1	6,4	6,2	10,2	6,5
LU	33,3	22,6	2,4	4,8	6,7	3,8
LV	47,6	33,2	15,0	9,8	5,4	3,6
MT	35,1	32,7	4,5	8,8	11,0	8,3
NL	17,4	13,9	5,1	3,6	7,0	1,8
PL	29,6	21,7	9,0	6,4	9,6	6,6
PT	30,2	25,5	4,5	4,6	8,0	7,3
RO	35,8	27,2	14,4	13,2	16,8	12,9
SE	8,4	7,3	1,7	0,7	1,5	2,7
SI	30,3	20,4	5,0	6,4	7,5	9,1
SK	20,5	18,3	13,5	14,3	11,1	12,0

Source: Eurobarometer, Gender Equality, March 2015. European Commission.

**Figure 8:**
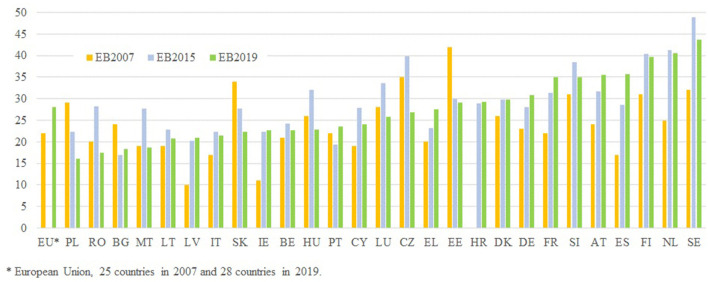
When a company wants to hire someone and has a choice between two candidates with the same skills and qualifications, in your opinion, does the candidate's gender put the candidate at a disadvantage? Source: Own elaboration from Eurobarometer, Discrimination in the European Union, 2007, 2015, and 2019.

To assess the perception of discrimination at work, this article examines the Eurobarometer surveys on Discrimination in the European Union from 2007, 2015, and 2019. Particularly, Figure 8 analyzes the perception of discrimination in access to positions through affirmative answers to the question: “When a company wants to hire someone and has a choice between two candidates with the same skills and qualifications, in your opinion, does the candidate's gender put the candidate at a disadvantage?” In general, it is observed that the perception of discrimination is higher in 2015 and 2019 than in 2007. Sweden, the Netherlands, Finland, Spain, Austria Slovenia, and France are highlighted as the countries with the highest perception of discrimination in 2019 whereas Poland, Romania, Bulgaria, and Malta are the countries with the lowest perception of discrimination. Interestingly, this is consistent with the fact that these are countries with very strong gender stereotypes, as can be seen in Table 4.

Thanks to the analysis of the data from the 2015 EWCS, Table 5 shows the percentage of men and women expressing disagreement or agreement with several statements. To make the chart easier to read, the cells in which the proportion of female responses is equal to or greater than male responses are shaded. The questions are the following:

**Table 5:** European working conditions survey 2015.

	**I receive the recognition I deserve for my work**		**My job offers good prospects for career advancement**		**Have you been subjected to discrimination at work in the last 12 months?**		**Are you treated fairly at your workplace?**	
	**Disagree**		**Disagree**		**Yes**		**Sometimes, rarely or never**	
	**Women**	**Men**	**Women**	**Men**	**Women**	**Men**	**Women**	**Men**
AT	**12**	10	**44**	33	**13**	12	10	11
BE	**15**	15	**43**	33	**10**	10	13	14
BG	14	19	37	37	2	3	12	16
CY	**10**	9	**30**	26	**9**	9	27	31
CZ	**12**	12	**34**	26	**6**	5	13	14
DE	**14**	14	**46**	35	**6**	6	9	10
DK	**11**	11	**24**	24	**7**	4	**9**	9
EE	12	17	32	33	9	11	12	14
EL	**14**	13	**37**	29	**10**	6	**19**	15
ES	16	17	**48**	42	**5**	5	21	22
FI	**12**	11	**38**	31	**11**	7	7	8
FR	**18**	16	**49**	40	**12**	10	**20**	18
HR	**29**	29	**47**	44	**4**	3	19	23
HU	**17**	17	28	32	4	7	16	18
IE	**18**	15	**36**	31	6	7	**14**	14
IT	**16**	14	**53**	42	**7**	6	13	19
LT	**17**	17	**45**	44	**6**	2	24	26
LU	**25**	18	**46**	32	**16**	11	**17**	10
LV	**22**	17	**37**	35	**8**	7	**18**	16
MT	18	22	30	33	**5**	4	16	19
NL	**12**	12	**43**	35	**13**	11	7	10
PL	**18**	21	27	28	2	3	27	28
PT	**12**	11	**38**	36	**5**	3	13	19
RO	**11**	8	**36**	25	8	9	**16**	15
SE	**15**	11	**41**	32	**14**	9	**14**	12
SI	**33**	30	**44**	38	**10**	6	18	19
SK	**31**	24	**45**	39	**8**	5	**34**	34
EU-27	**16**	15	**41**	35	**8**	7	15	17

Source: Own elaboration from EWCS (2015). The bold values indicate that these are the situations in which women feel more affected than men.

“I receive the recognition I deserve for my work”: In most countries, women disagree with this information to a greater extent than men. The figure is particularly high in Slovakia, Slovenia, and Hungary where nearly one-third of the women surveyed feel that they do not receive the recognition they deserve.

“My job offers good prospects for career advancement”: There is a generalized disagreement in all countries, and it is higher among women than men, especially in Italy, Luxembourg, Romania, Germany, and Austria.

“Have you been subjected to discrimination at work in the last 12 months?”: Women have a higher perception of discrimination toward themselves than men. This is most notable in Luxembourg, Sweden, the Netherlands, and Austria.

“Are you treated fairly at your workplace?”: There is not a substantial difference between men and women. However, the proportion of people who think they are treated fairly on rare occasions at their workplace is very high in Slovakia, Cyprus, Poland, and Lithuania.

**Figure 9:**
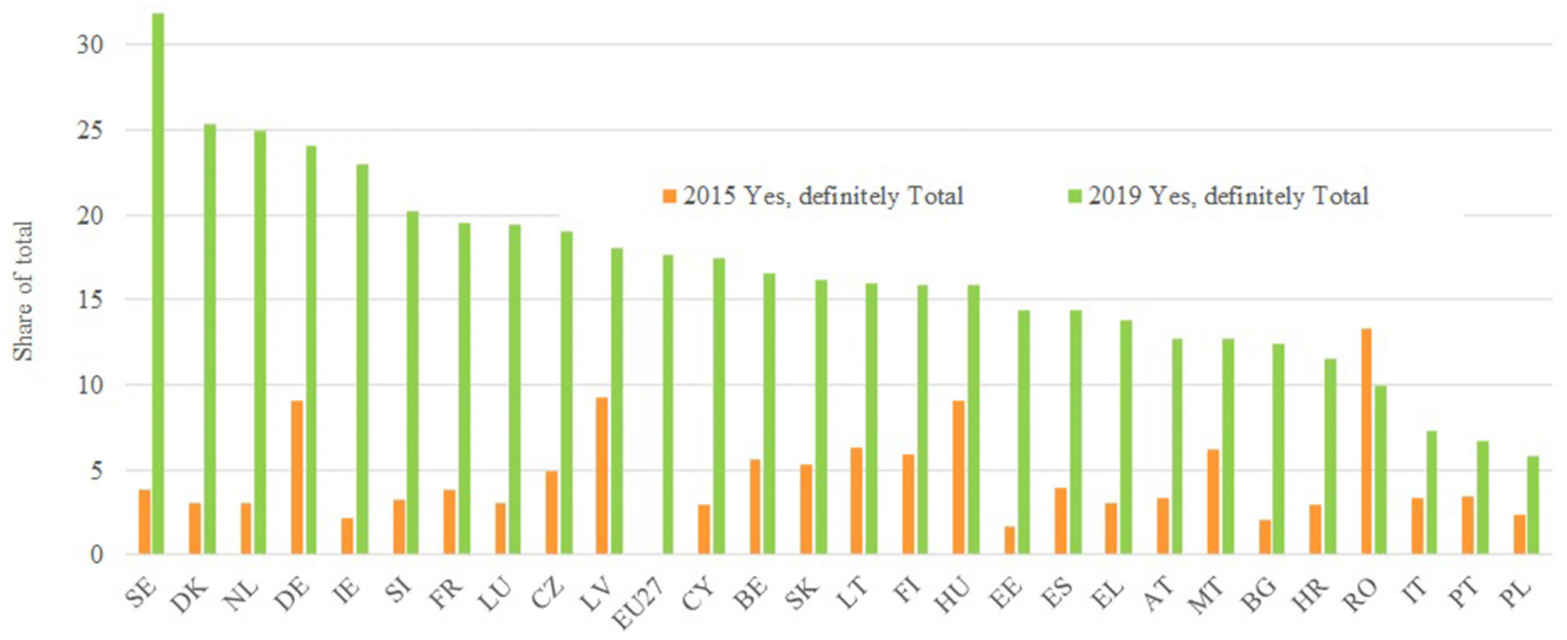
Is enough being done to promote gender equality in your workplace? Source: Own elaboration from Eurobarometer, Discrimination in the European Union, 2015 and 2019.

Finally, Figure 9 shows the percentages of affirmative responses to the question: “Is enough being done to promote diversity in your workplace?” This question is relevant, for it illustrates how employees perceive the efforts of their respective companies to promote gender equality and what are the changes in corporate culture in this regard.

The data show that the change in workers' perception of the effort being made in their respective workplaces is very high. That is, workers in all countries, except for Romania, acknowledge that more is being done in their workplaces to promote gender equality. Remarkably, however, it is not the specific percentage of each country, but the fact of recognizing that inequality exists and that companies have a role to play in solving the problem.

## 6. Discussion

Although women's participation in the labor market has significantly grown in recent years, the glass ceiling prevents women from occupying all positions in the organizational hierarchy on an equal level to men. For this reason, several regulations, both at the community and state levels, have been developed to promote the achievement of equal opportunities between men and women in the business environment.

Key strategies in the fight against gender inequality in companies have been CSR and GSR which, respectively, aim to have a clear influence on the cultural patterns of organizations. In the last few decades, certain interest groups have demanded the incorporation of the concept of gender mainstreaming into business policies since before acting, it is necessary to define and reflect on which stakeholders will be affected by the company's decision. It is against this backdrop that we believe that there is a necessity to add sustainable development to the corporate objective of value creation, for it would ensure a balance between economic growth and social welfare. As it has been found, corporate culture cannot be legislated as such which is why the EU had to create binding regulations. These rules have been aimed at forcing organizations not to group economic power in men justified exclusively by a biological reason.

After the examination of the European legislation on equality, it could be asserted that progress is slow since the data reveal that if current trends continue, we will need almost another 100 years to achieve full equality. Within the sections in which gender discrimination in the workplace is usually analyzed, it can be claimed that the ones that still require the greatest effort are money (pay gaps), time (precariousness of women's working conditions and family life balance), and power (presence in decision-making). Particularly, the present study has focused on dissecting the last sector which corresponds to the presence of women in corporate decision-making bodies. This has revealed that the most effective measure for the underrepresentation of women in the sphere of corporate power is the so-called “minimum share” since countries that have adopted gender parity laws on boards of directors have increased the presence of women in them.

Despite regulatory efforts, statistical analysis shows that the situation of women in the labor market continues to be discriminatory: They are the ones who have lower activity rates, higher unemployment rates, lower salaries, and live with the glass ceiling that prevents them from reaching decision-making positions. However, we contend that there are still weaknesses within the EU legislative framework since not all measures are binding and those that are binding normally have limited coercive capacity, that is, as there are practically no sanctions for non-compliance with the standard, most of the texts are based on a series of recommendations that do not detail how to implement effective equality measures in companies.

As far as the quantitative analysis of the business climate is concerned, this has been extremely complex since it includes many qualitative elements that cannot be captured in observable variables. Thus, in the absence of any European statistics on business culture and its impact on gender inequality, we have conducted the research using labor statistics and opinion surveys. Remarkably, however, the results on the proportion of women in the highest corporate decision-making body show a very regular pattern of behavior. Namely, in the early 2000s, the presence of women in these bodies was practically absent, whereas, in the 2010s, there is a change in the trend due to the legal measures imposed for equality between women and men, proving that these have been effective.

In sum, if the maintenance of this inclination is desired, there needs to be a transformation along with a cultural change in the company: Gender equality must be recognized by organizations as an internal moral obligation beyond any legal text. It is necessary to adapt the organizational culture to the new social situation capable of overcoming values based on gender stereotypes.

## Data availability statement

The raw data supporting the conclusions of this article will be made available by the authors, without undue reservation.

## Author contributions

Both authors developed, discussed, worked on the article, the legal analysis has been carried out mainly by IG and the statistical analysis mainly by NA. Both authors contributed to the article and approved the submitted version.

## Footnotes

^1^The European Enterprise Survey (ECS) has been carried out regularly since its inception in 2004–2005 as the European Establishment Survey on Working Time and Work-Life Balance (ESWT), but, unfortunately, none of the items of the survey applies to our analysis.

^2^“Eurobarometer is the polling instrument used by the European Commission, the European Parliament, and other EU institutions and agencies to regularly monitor the state of public opinion in Europe on issues related to the European Union as well as attitudes on subjects of political or social nature. Eurobarometer provides quality and relevant data for experts in public opinion, researchers, media, and the public.”

^3^ https://eige.europa.eu/gender-equality-index

^4^The European Company Survey (ECS), conducted jointly by Eurofound and Cedefop in 2004, 2009, 2013, and 2019, provides information on workplace practices in terms of work organization, human resources management, and so on. Nonetheless, it neither includes any items on the position of women in the company nor the subdivision of responses between men and women.

^5^The nomenclature of the EU countries corresponds to abbreviations of the names in the national language of each of them; the alphabetical order of the abbreviations is as follows: Austria AT, Belgium BE, Bulgaria BG, Cyprus CY, Czechia CZ, Germany DE, Denmark DK, Estonia EE, Greece EL, Spain ES, European Union - 27 countries EU-27, Finland FI, France FR, Croatia HR, Hungary HU, Ireland IE, Italy IT, Lithuania LT, Luxembourg LU, Latvia LV, Malta MT, Netherlands NL, Poland PL, Portugal PT, Romania RO, Sweden SE, Slovakia SK, and Slovenia SL.

^6^The wage gap used is called the unadjusted gender pay gap and is defined by Eurostat as the difference between the average gross hourly earnings of men and women expressed as a percentage of the average gross hourly earnings of men. It is usually calculated for enterprises with 10 or more employees.

^7^The EWCS is developed every 5 years. However, the 2020 edition was not carried out due to the pandemic and that is why, in 2021, it was elaborated using an extraordinary telephone edition in which specific questions on working conditions during COVID-19 were included.
